# Preferred Practice Patterns of Congenital Nasolacrimal Duct Obstruction in Jordan

**DOI:** 10.2147/OPTH.S421054

**Published:** 2023-08-11

**Authors:** Hashem Abu Serhan, Jehad Feras AlSamhori, Abdelmonem Siddiq, Abdul Rhman Hassan, Sara Irshaidat, Leen Abu Serhan, Abdullah Alawadhi, Abdelaziz Abdelaal, Wejdan Al-Thawabieh

**Affiliations:** 1 Department of Ophthalmology, Hamad Medical Corporations, Doha, Qatar; 2 Faculty of Medicine, University of Jordan, Amman, Jordan‬‬‬‬‬‬; 3 Faculty of Pharmacy, Mansoura University, Mansoura, Egypt; 4 Department of Ophthalmology, Visual and Anatomical Sciences, Wayne State University School of Medicine, Detroit, MI, USA; 5 Department of Pediatrics, King Hussein Cancer Center, Amman, Jordan; 6 Faculty of Medicine, Hashemite University, Zarqa, Jordan; 7 Department of Ophthalmology, Islamic Hospital, Amman, Jordan; 8 Harvard Medical School, Postgraduate Medical Education, Boston, MA, USA; 9 Department of Ophthalmology, Dr. Sulaiman Al Habib Hospital, Riyadh, Saudi Arabia

**Keywords:** nasolacrimal duct obstruction, CNLDO, lacrimal system, practice patterns, congenital

## Abstract

**Purpose:**

Congenital nasolacrimal duct obstruction (CNLDO) is fairly common in newborns. The main aim of this cross-sectional study is to assess the preferred practice patterns of CNLDO among ophthalmologists in Jordan.

**Methods:**

This cross-sectional study was conducted across all ophthalmological practices in Jordan, using convenience sampling. An online questionnaire, designed through Google Forms, was distributed through social media. The survey contained four domains: baseline characteristics of participants and the diagnosis (7 items), medical management (3 items), and surgical management (11 items) of CNLDO. Descriptive statistics were conducted using SPSS (IBM SPSS Corp, SPSS Statistics ver. 26, USA).

**Results:**

Eighty-three physicians responded to the survey, with an average age of 40.6 ± 8.6. More than half of the participants (53.0%, n = 44) were general ophthalmologists. Only 37.3% of our sample (n = 31) regularly evaluated the refraction of a child presenting with epiphora suggestive of CNLDO. Criggler’s nasolacrimal duct massage was recommended by 62.7% of respondents (n = 52) for up to 12 months. In addition, 72.3% of respondents (n = 60) recommended 12 months as the minimum age for primary probing of CNLDO. Silicon intubation was considered for primary probing starting at 24 months by 31.3% of ophthalmologists (n = 20). Monocanalicular stent was preferred by 42.2% of respondents (n = 27) while 31.3% (n = 20) preferred bicanalicular stent.

**Conclusion:**

There is considerable variability in preferred practice patterns regarding the diagnosis and management of CNLDO in Jordan. Our findings highlight the gaps in optimum practices which need to be addressed for better management.

## Introduction

Congenital nasolacrimal duct obstruction (CNLDO) is a common disorder among children occurring in 6–20% of children with 80% of the cases are unilateral.^1^ The pathogenesis of the CNLDO is due to mechanical membranous obstruction at the valve of Hasner due to failure of recanalization, just before the nasolacrimal duct inserts in the nasal cavity.^2^ The mechanical obstruction could be also due to facial bone abnormality or stenosis of the distal part of the nasolacrimal duct at the inferior meatus.^3^ Genetic predisposition, intrapartum infection, prematurity, and exposure to radiation or certain medications are some of the risk factors for CNLDO.^4^,^5^ The nasolacrimal duct is essential for the drainage of tears. Tears are synthesized in the lacrimal gland with the physiological function of lubricating the eyes. They are normally drained from the lacrimal sac to the nasal cavity through the nasolacrimal duct at the inferior meatus of the nasal cavity. Therefore, CNLDO leads to an abnormal overflow of tears onto the face termed epiphora.^6^ Infections could occur on the top of CNLDO, presenting with mucinous discharge, eyelashes crusting, and eyelid dermatitis.^7^ Furthermore, the persistence of CNLDO can lead to chronic preseptal and orbital cellulitis as well as dacryocystitis.^8^

Diagnosis of CNLDO is clinical and can be confirmed using the fluorescein dye disappearance test.^9^ During their first year of life, 80–90% of CNDO resolve either spontaneously or with conservative management.^10^ Probabilities of spontaneous resolution by month until 12 months of age are 80–90% at 3 months of age, 68–75% at 6 months of age, and 36–57% at 9 months of age.^11^ Conservative measures include massaging the lacrimal sac using Criggler’s technique as well as antibiotics in the presence of bacterial infection of the stagnant tears. When conservative measures fail, probing of nasolacrimal duct is the first line of management. In children younger than 18 months old, success rates of 77% to 97% have been reported.^12^ However, the optimal timing of probing remains controversial. The main problem is that spontaneous resolution can occur after 13 months of age. Rates of spontaneous resolution of CNDO at 13–24 months of age are 60.0–79.3%. In addition, spontaneous resolution of CNDO with conservative therapies can occur up to 48 months of age. On the other hand, Young et al^13^ reported that the cure rate for cases with probing performed at 12–24 months of age was significantly higher than that for spontaneous resolution. Other available interventions are balloon catheter dilation and duct intubation, and external or endoscopic dacryocystorhinostomy (DCR).^14^,^15^

The main aim of this cross-sectional study is to assess the preferred practice patterns of CNLDO among ophthalmologists in Jordan. Building a consensus on the preferred management paradigm of CNLDO will help both physicians and patients. To our knowledge, this is the first study evaluating the preferred practice patterns of CNLDO among ophthalmologists in Jordan.

## Methods

### Study Design and Participants

This is a descriptive, cross-sectional study that was conducted using an online questionnaire targeting all ophthalmologists practicing in Jordan including public, private, and university hospitals. We followed the Checklist for Reporting Results of Internet E-Surveys (CHERRIES) in conducting the questionnaire (Appendix 1).^16^ Data was collected using web-based questionnaire software (Google forms). Participants were recruited through social media (E-mail, Facebook, and WhatsApp) by sending an invitation to complete the questionnaire after providing a brief description of the study from September to December 2022. Participants were informed before starting the questionnaire that it is completely anonymous, and voluntary and that all data would be treated as confidential.

### Items in the Survey

A 20-minute online survey on Google form that was based on current scientific literature by referring to the literature and included main ideas and primary items relevant to our topic was used in this study. The face and content validity of the questionnaire was established by reviewing the questionnaire by 5 experts in the field who provided their feedback and suggested necessary changes. The reliability of the questionnaire was established through pilot testing by collecting data from 20 general ophthalmologists who were asked to provide their feedback and these were not included in the study sample. The questionnaire was completely anonymous and did not contain any identifying information. To ensure that it was impossible to link responses with their providers, the Internet protocol address was omitted from individual responses at the time of analysis.

The survey consisted of four attributes. The first section is for demographic data of the participants who were asked to report their age, gender, specialty, years of experience, and type of practice. The second section included seven questions on the diagnostic approach of CNLDO. The third section included three questions investigating the medical management of CNLDO including Criggler’s massage, and probing. The last section included 11 questions targeting the surgical management of CNLDO including in-fracture of the inferior turbinate, silicon intubation, balloon catheter dilatation, and DCR. The questionnaire items are included in Appendix 2.

### Sample Size and Sampling Technique

The sample size was calculated using epiinfoTM v.7.2.4.0 (epiinfoTM, version 7.2.4.0, a database and statistics program for public health professionals. CDC, Atlanta, GA, USA, 2011) considering a previous study performed in Jordan with an applied reliability of 0.749 (ie, Cronbach’s alpha).^17^ Using a cross-sectional study design, where n = required sample size (n = Z (α/2) 2 pq/d2), we calculated the sample size based on the following parameters:

Prevalence of 480 registered ophthalmologists,^18^ Cronbach’s alpha of 0.749, 99.99% confidence interval (CI), and 5% margin of error. We estimated 41 as the minimum sample size required to represent the true population. A total of 83 subjects were included in this study.

### Data Analysis

The information was taken from Google Forms and converted to an Excel spreadsheet before being entered into the Statistical Package for Social Sciences (SPSS) version 27 (IBM SPSS Corp, SPSS Statistics ver. 26, USA). Descriptive analysis was used to display categorical variables as percentages and frequencies while presenting numerical variables as a mean and standard deviation to evaluate the data quantitatively. The significance of the data was determined using a categorical Chi-square test. All statistical tests were conducted with a 95% confidence interval and a 5% error margin. A p-value of less than 0.05 was considered statistically significant.

### Ethical Approval

The Institutional Review Board committee of the Islamic Hospital, Amman, Jordan, considered this study to be exempt from IRB review and approval. All study procedures were conducted according to the principles of the World Medical Association Declaration of Helsinki and its amendments.^19^

## Results

### Demographic Characteristics of Participants

Eighty-three physicians responded to the survey, with an average age of 40.6 ± 8.6. Males made up 63.9% (n = 53) of the total, while female respondents made up 36.1% (n = 30). The majority of participants (61.4%, n = 61) had less than ten years of experience. More than half of the participants (53.0%, n = 44) were general ophthalmologists. Thirty-six of them (43.4%) worked in private practice. Table 1 shows the demographic characteristics of participants.

**Table 1:** Demographic Characteristics of the Participants

Item	Frequency (%)
Age	40.6 ± 8.6
Gender	
Male	53 (63.9%)
Female	30 (36.1%)
How many years have you been in practice?	
5 years	29 (34.9%)
5–10 years	22 (26.5%)
11–15 years	10 (12.0%)
16–20 years	11 (13.3%)
>20 years	11 (13.3%)
What is your specialty?	
General ophthalmologist	44 (53.0%)
Pediatric ophthalmologist	18 (21.7%)
Oculoplastic ophthalmologist	9 (10.8%)
Vitreoretinal ophthalmologist	7 (8.4%)
Neuro-ophthalmologist	2 (2.4%)
Glaucoma	1 (1.2%)
Anterior Segment	1 (1.2%)
Cornea	1 (1.2%)
Type of practice?	
Ministry of Health	25 (30.1%)
University hospitals	9 (10.8%)
Private practice	36 (43.4%)
Military hospitals	13 (15.7%)

### Diagnostic Approach of Congenital Nasolacrimal Duct Obstruction

For a child presenting with epiphora suggestive of CNLDO, 49.4% of ophthalmologists (n = 41) used fluorescein dye to estimate the lacrimal lake and confirm the diagnosis. Chi-square test was conducted to evaluate the association between the usage of fluorescein dye and the usage of Dye Disappearance test (DDT) in the diagnosis of CNLDO. It was found that physicians who always used fluorescein dye would use DDT in aiding the diagnosis. (x^2^= 63.34, p-value <0.001). Furthermore, 50.6% of ophthalmologists (n = 42) inspect the inferior puncta at least to check for patency of lacrimal puncta for a child presenting with epiphora. Only 37.3% of our sample (n = 31) regularly evaluated the refraction of a child presenting with epiphora suggestive of CNLDO. Almost 44.6% of ophthalmologists (n = 37) would consider congenital glaucoma as one of the differential diagnoses for a child with epiphora. Table 2 shows the preferred practice patterns of the diagnostic approach of CNLDO.

**Table 2:** Preferred Practice Patterns of the Diagnostic Approach of CNLDO

Item	Frequency (%)
For a child presenting with epiphora suggestive of CNLDO, how often do you use fluorescein dye to estimate lacrimal lake?	
I always use it	41 (49.4%)
Sometimes	25 (30.1%)
I do not use it and rely mostly on inspection of unstained lacrimal lake	17 (20.5%)
The following response best describes your use of Dye disappearance test in the diagnosis of CNLDO:	
I use it and wait for 3 minutes before considering it positive for obstruction	12 (14.5%)
I use and wait for 4 minutes to conclude	4 (4.8%)
I use it and wait for 5 minutes to conclude	44 (53.0%)
I do not use it and I rely mostly on the clinical picture to conclude the diagnosis of CNLDO	21 (25.3%)
I use it and wait for 10 minutes to conclude	2 (2.4%)
Do you make a conscious effort to check for patency of lacrimal puncta for a child presenting with epiphora?	
Yes, I try to inspect all four puncta for every patient if possible	31 (37.3%)
Yes, I try to inspect the inferior puncta at least if possible	42 (50.6%)
Yes, sometimes and depending on the clinical picture	10 (12.0%)
No, I do not pay attention to that	0 (0.0%)
One of the following patients is in more need for inspection of the puncta as part of examination and evaluation done in the clinic:	
A child with epiphora and clear tearing but no discharge or no sticky lashes	56 (67.5%)
A child with epiphora and turbid discharge and sticky lashes	27 (32.5%)
How often do you check for reflux at the lacrimal puncta for a child presenting with epiphora suggestive of CNLDO?	
I always do it. I think it is necessary	49 (59.0%)
Sometimes and depending on the clinical picture	27 (32.5%)
I do not think it is necessary by any means	7 (8.5%)
Regarding refraction of a child presenting with epiphora suggestive of CNLDO. My preference is:	
I always make sure to test properly with wet refraction	31 (37.3%)
Just dry or auto-refraction if possible	17 (20.5%)
I do not examine refraction regularly	35 (42.2%)
One of the following responses best describes your attitude towards dealing with congenital glaucoma as one of the differential diagnoses for epiphora in the young age group:	
I always check IOP at least digitally and look at optic nerves for all children presenting with epiphora	37 (44.6%)
I consider it if the child has obvious signs such as enlarged globe or hazy cornea	44 (53.0%)
I am not aware of this association usually	2 (2.4%)

### Medical Management of Congenital Nasolacrimal Duct Obstruction

Criggler’s nasolacrimal duct massage was recommended by 62.7% of respondents (n = 52) for up to 12 months. In addition, 72.3% of respondents (n = 60) recommended 12 months as the minimum age for primary probing of CNLDO. In terms of surgical management of CNLDO, 37.3% of ophthalmologists (n = 31) would not treat CNLDO surgically. However, they would prefer to refer them for probing at or before the age of 18 months. Table 3 shows the preferred practice patterns of medical management of CNLDO.

**Table 3:** Preferred Practice Patterns of Medical Management of CNLDO

Item	Frequency (%)
Regarding Criggler’s massage of the nasolacrimal duct, the following response best describes your preferred practice pattern:	
I recommend it up to 6 months	5 (6.0%)
I recommend it up to 9 months	14 (16.9%)
I recommend it up to 12 months	52 (62.7%)
I would give it a try even if the child presents later than 18 months	9 (10.8%)
I do not recommend it or emphasize it enough to the parents	3 (3.6%)
What is the minimum age that you would recommend for probing/ surgical management of CNLDO in your practice (apart from probing done after bout(s) of dacryocystitis which should be dealt with at a younger age)?	
9 to 10 months before I would consider referral or treatment	19 (22.9%)
12 months	60 (72.3%)
18 months	4 (4.8%)
24 months	0 (0.0%)
Regarding surgical management of children presenting with a picture of CNLDO:	
I do not surgically treat children with CNLDO. I refer them at or before the age for probing	31 (37.3%)
I do probe and surgical management myself if needed	52 (62.7%)

### Surgical Management of Congenital Nasolacrimal Duct Obstruction

Simple probing was preferred as the first intervention for a child presenting with CNLDO by 39.1% of respondents (n = 25) at the age of 12 months. Most of respondents (64.1%, n = 41) do not have the experience to do infracture of inferior turbinate while 17.2% of them (n = 11) do it for cases with failed probing. Silicon intubation was considered for primary probing starting at 24 months by 31.3% of ophthalmologists (n = 20). Monocanalicular stent was preferred by 42.2% of respondents (n = 27) while 31.3% (n = 20) preferred bicanalicular stent. If silicon intubation (stent) was used during the procedure, 50.0% of them (n = 32) would wait 3 months before removal of stent while 37.5% (n = 24) would wait for 6 months to remove it. For the child presenting with epiphora suggestive of CNLDO with no history of any intervention, 40.6% (n = 26) of respondents would never perform DCR as a primary procedure. In case of a failed primary simple probing, 42.2% of them (n = 27) would repeat probing with silicon tube stenting regardless of the age, on contrary, 23.4% of them (n = 15) would refer failed cases to oculoplastic or pediatric ophthalmologists. Table 4 shows the preferred practice patterns of surgical management of CNLDO.

**Table 4:** Preferred Practice Patterns of Surgical Management of CNLDO

Item	Frequency (%)
Simple probing is your preferred choice as the first intervention for the child presenting with CNLDO up to what age?	
12 months	25 (39.1%)
18 months	0 (0.0%)
24 months	23 (35.9%)
3 years	4 (6.2%)
4 years	0 (0.0%)
I would always consider it as a first procedure regardless of age	12 (18.8%)
The following statement best describes your experience with infracture of inferior turbinate:	
I never do it	41 (64.1%)
Only for cases with failed probing	11 (17.2%)
I do it sometimes for selected cases	12 (18.8%)
You would consider silicon intubation for primary probing starting at what age at presentation?	
12 months	6 (9.4%)
18 months	0 (0.0%)
24 months	20 (31.2%)
3 years	9 (14.1%)
4 years	0 (0.0%)
I would not consider it for primary probing at any age. I prefer simple probing first	16 (25.0%)
I have no experience with silicon intubation and I usually refer if I suspect its need	13 (20.3%)
Your preferred type of stenting is:	
Monocanalicular	27 (42.2%)
Bicanalicular	20 (31.3%)
I do not use them	17 (26.5%)
If a child is stented during the procedure, how long would you leave it in place before consider removal of stent/ tube?	
1 month	3 (4.7%)
2 months	4 (6.3%)
3 months	32 (50.0%)
6 months	24 (37.5%)
9 months	1 (1.6%)
12 months	0 (0.0%)
A child is scheduled for removal of silicon tube. What is your preferred practice?	
Office with topical anesthesia whenever possible	40 (62.5%)
OR with sedation or general anesthesia	24 (37.5%)
The following statement best describes your experience with Balloon Catheter dilatation:	
I am skeptical with regard to its added value	20 (31.3%)
I usually use it with primary probing if clinical picture suggests narrow duct rather than full obstruction	4 (6.3%)
I would use it only for failed and redo probing	7 (10.8%)
I would like to consider their use but not available where I practice	33 (51.6%)
The following statement best describes your experience with Dacryocystorhinostomy (DCR):	
I have no experience doing it	40 (62.5%)
I do DCR. External is my preferred choice	20 (31.3%)
Yes, Endoscopic endonasal DCR is my preferred choice if available	4 (6.3%)
Yes, Non-endoscopic endonasal DCR	0 (0.0%)
Yes, Laser DCR	0 (0.0%)
A child presents to you with epiphora suggestive of CNLDO with no history of any intervention. When would you consider DCR for the primary procedure?	
If age >3 years	5 (7.8%)
If age >4 years	6 (9.4%)
If age >6 years (neglected cases)	3 (4.7%)
I would never do it as a primary intervention	26 (40.6%)
I would not do it myself but would consider for selected cases of failed probing	24 (37.5%)
The following statement best describes your experience regarding the child presenting with failed primary simple probing:	
Repeat simple probing keeping in mind the age limit that I stick to with regard to stent placement	18 (28.1%)
Repeat probing with silicon tube stenting regardless of age	27 (42.2%)
Repeat probing with infracture of inferior turbinate ± silicon intubation	4 (6.3%)
Refer failed cases to oculoplastic and pediatric ophthalmologists	15 (23.4%)
The following statement best describes your experience with the child presenting with failed probing and stenting:	
Redo probing with stenting	10 (15.6%)
Redo probing with stenting with infracture of inferior turbinate	8 (12.5%)
Redo with DCR done by myself	7 (10.9%)
Refer to a colleague who does DCR	39 (60.9%)

### Effect of Specialty on the Preferred Practice Patterns of Congenital Nasolacrimal Duct Obstruction

Chi-square test was conducted to assess the effect of the specialty of the participants as general or pediatric/specialized ophthalmologists on their preferred practice patterns of CNLDO. For a child presenting with epiphora suggestive of CNLDO, there was significant difference between the practice of general and pediatric ophthalmologists. Pediatric ophthalmologists (54.2%, n = 13) were more capable to test refraction than general ophthalmologists (45.8%, n = 11) (p-value= 0.002). Furthermore, 80% of general ophthalmologists (n = 36) would recommend probing/surgical management of CNLDO at 12 months, on the other hand, only 20% of pediatric ophthalmologists (n = 9) would recommend it at 12 months (p-value=0.03). For a CNLDO with failed primary simple probing, 57.1% of general ophthalmologists (n = 8) would prefer repeating simple probing in comparison to 42.9% of pediatric ophthalmologists (n = 6) (p-value= 0.010). When comparing between general and specialized ophthalmologists in their preferred practice with infracture of inferior turbinate, 58.5% of general ophthalmologists stated that they never done it before (n = 24) while 41.5% of specialized ophthalmologists did not (n = 17) (p-value= 0.014). Table 5 shows the effect of specialty on the preferred practice patterns of CNLDO.

**Table 5:** The Effect of Specialty on the Preferred Practice Patterns of CNLDO

Item	Specialty		P-value	Specialty		P-value
	General Ophthalmologist	Pediatric Ophthalmologist		General Ophthalmologist	Specialized Ophthalmologist	
For the child presenting with epiphora suggestive of CNLDO, how often do you use fluorescein dye to estimate lacrimal lake?			0.439			0.416
I always use it	19 (63.3%)	11 (36.7%)		19 (46.3%)	22 (53.7%)	
Sometimes	14 (77.8%)	4 (22.2%)		14 (56.0%)	11 (44.0%)	
I do not use it and rely mostly on inspection of unstained lacrimal lake	11 (78.6%)	3 (21.4%)		11 (64.7%)	6 (35.3%)	
The following response best describes your use of Dye Disappearance test (DDT) in the diagnosis of CNLDO:			0.228			0.483
I use it and wait for 3 minutes before considering it positive for obstruction	7 (70.0%)	3 (30.0%)		7 (58.3%)	5 (41.7%)	
I use and wait for 4 minutes to conclude	1 (25.0%)	3 (75.0%)		1 (25.0%)	3 (75.0%)	
I use it and wait for 5 minutes to conclude	21 (72.4%)	8 (27.6%)		21 (47.7%)	23 (52.3%)	
I do not use it and I rely mostly on the clinical picture to conclude the diagnosis of CNLDO	14 (82.4%)	3 (17.6%)		14 (66.7%)	7 (33.3%)	
I use it and wait for 10 minutes to conclude	1 (50.0%)	1 (50.0%)		1 (50.0%)	1 (50.0%)	
Do you make a conscious effort to check for patency of lacrimal puncta for a child presenting with epiphora?			0269			0.155
Yes, I try to inspect all four puncta for every patient if possible	15 (65.2%)	8 (34.8%)		15 (48.4%)	16 (51.6%)	
Yes, I try to inspect the inferior puncta at least if possible	0 (0.0%)	0 (0.0%)		26 (61.9%)	16 (38.1%)	
Yes, sometimes and depending on the clinical picture	26 (78.8%)	7 (21.2%)		3 (30.0%)	7 (70.0%)	
No, I do not pay attention to that	3 (50.0%)	3 (50.0%)		44 (53.0%)	39 (47.0%)	
One of the following patients is in more need for inspection of the puncta as part of examination and evaluation done in the clinic:			0.633			0.278
A child with epiphora with clear tearing and no discharge and no sticky lashes	32 (72.7%)	12 (27.3%)		32 (57.1%)	24 (42.9%)	
A child with epiphora with turbid discharge and sticky lashes	12 (66.7%)	6 (33.3%)		12 (44.4%)	15 (55.6%)	
How often do you check for reflux at the lacrimal puncta for the child presenting with epiphora suggestive of CNLDO?			0.830			0.555
I always do it. I think it is necessary	28 (71.8%)	11 (28.2%)		28 (57.1%)	21 (42.9%)	
Sometimes and depending on the clinical picture	12 (66.7%)	6 (33.3%)		12 (44.4%)	15 (55.6%)	
I do not think it is necessary by any means	4 (80.0%)	1 (20.0%)		4 (57.1%)	3 (42.9%)	
Regarding refraction of the child presenting with epiphora suggestive of CNLDO. My preference is:			0.002			0.047
I always make sure to test properly with wet refraction	11 (45.8%)	13 (54.2%)		11 (35.5%)	20 (64.5%)	
Just dry or auto-refraction if possible	11 (78.6%)	3 (21.4%)		11 (64.7%)	6 (35.3%)	
I do not examine refraction regularly	22 (91.7%)	2 (8.3%)		22 (62.9%)	13 (37.1%)	
One of the following responses best describes your attitude towards dealing with congenital glaucoma as one of the differential diagnoses for epiphora in the young age group:			0.184			0.265
I always check IOP at least digitally and look at optic nerves for all children presenting with epiphora	16 (59.3%)	11 (40.7%)		16 (43.2%)	21 (56.8%)	
I consider it if the child has obvious signs such as enlarged globe or hazy cornea	27 (79.4%)	7 (20.6%)		27 (61.4%)	17 (38.6%)	
I am not aware of this association usually	1 (100.0%)	0 (0.0%)		1 (50.0%)	1 (50.0%)	
Regarding Criggler’s massage of the nasolacrimal duct, the following response best describes your preferred practice pattern:			0.413			0.407
I recommend it up to 6 months	4 (100.0%)	0 (0.0%)		4 (80.0%)	1 (20.0%)	
I recommend it up to 9 months	9 (75.0%)	3 (25.0%)		9 (64.3%)	5 (35.7%)	
I recommend it up to 12 months	26 (68.4%)	12 (31.6%)		26 (50.0%)	26 (50.0%)	
I would give it a try even if the child presents later than 18 months	3 (50.0%)	3 (50.0%)		3 (33.3%)	6 (66.7%)	
I do not recommend it or emphasize it enough to the parents	2 (100.0%)	0 (0.0%)		2 (66.7%)	1 (33.3%)	
What is the minimum age that you would recommend for probing/ surgical management for CNLDO in your practice (apart from probing done after bout(s) of dacryocystitis which should be dealt with at a younger age)?			0.033			0.109
9 to 10 months before I would consider referral or treatment	7 (50.0%)	7 (50.0%)		7 (36.8%)	12 (63.2%)	
12 months	36 (80.0%)	9 (20.0%)		36 (60.0%)	24 (40.0%)	
18 months	1 (33.3%)	2 (66.7%)		1 (25.0%)	3 (75.0%)	
24 months	0 (0.0%)	0 (0.0%)		0 (0.0%)	0 (0.0%)	
Regarding surgical management of children presenting with a picture of CNLDO:			0.015			0.243
I do not surgically treat children with CNLDO. I refer them at or before the age for probing	19 (90.5%)	2 (9.5%)		19 (61.3%)	12 (38.7%)	
I do probe and surgical management myself if needed	25 (61.0%)	16 (39.0%)		25 (48.1%)	27 (51.9%)	
Simple probing is your preferred choice as the first intervention for the child presenting with CNLDO up to what age?			0.313			0.315
12 months	11 (64.7%)	6 (35.3%)		11 (44.0%)	14 (56.0%)	
18 months	0 (0.0%)	0 (0.0%)		0 (0.0%)	0 (0.0%)	
24 months	14 (66.7%)	7 (33.3%)		14 (60.9%)	9 (39.1%)	
3 years	1 (33.3%)	2 (66.7%)		1 (25.0%)	3 (75.0%)	
4 years	0 (0.0%)	0 (0.0%)		0 (0.0%)	0 (0.0%)	
I would always consider it as a first procedure regardless of age	8 (88.9%)	1 (11.1%)		8 (66.7%)	4 (33.3%)	
The following statement best describes your experience with infracture of inferior turbinate:			0.050			0.014
I never do it	24 (72.7%)	9 (27.3%)		24 (58.5%)	17 (41.5%)	
Only for cases with failed probing	8 (80.0%)	2 (20.0%)		8 (72.7%)	3 (27.3%)	
I do it sometimes for selected cases	2 (28.6%)	5 (71.4%)		2 (16.7%)	10 (83.3%)	
You would consider silicon intubation for primary probing starting at what age at presentation?						0.463
12 months	3 (75.0%)	1 (25.0%)		3 (50.0%)	3 (50.0%)	
18 months	0 (0.0%)	0 (0.0%)		0 (0.0%)	0 (0.0%)	
24 months	8 (50.0%)	8 (50.0%)		8 (40.0%)	12 (60.0%)	
3 years	4 (57.1%)	3 (42.9%)		4 (44.4%)	5 (55.6%)	
4 years	0 (0.0%)	0 (0.0%)		0 (0.0%)	0 (0.0%)	
I would not consider it for primary probing at any age. I prefer simple probing first	10 (71.4%)	4 (28.6%)		10 (62.5%)	6 (37.5%)	
I have no experience with silicon intubation, and I usually refer if I suspect its need	9 (100.0%)	0 (0.0%)		9 (69.2%)	4 (30.8%)	
Your preferred type of stenting is:			0.069			0.072
Monocanalicular	10 (50.0%)	10 (50.0%)		10 (37.0%)	17 (63.0%)	
Bicanalicular	12 (75.0%)	4 (25.0%)		12 (60.0%)	8 (40.0%)	
I do not use them	12 (85.7%)	2 (14.3%)		12 (70.6%)	5 (29.4%)	
If a child is stented during the procedure, how long would you leave it in place before consider removal of stent/ tube?			0.862			0.848
1 month	2 (66.7%)	1 (33.3%)		2 (66.7%)	1 (33.3%)	
2 months	2 (50.0%)	2 (50.0%)		2 (50.0%)	2 (50.0%)	
3 months	17 (68.0%)	8 (32.0%)		17 (53.1%)	15 (46.9%)	
6 months	13 (72.2%)	5 (27.8%)		13 (54.2%)	11 (45.8%)	
9 months	0 (0.0%)	0 (0.0%)		0 (0.0%)	1 (100.0%)	
12 months	0 (0.0%)	0 (0.0%)		0 (0.0%)	0 (0.0%)	
A child is scheduled for removal of silicon tube. What is your preferred practice?			0.054			0.093
Office with topical anesthesia whenever possible	18 (58.1%)	13 (41.9%)		18 (45.0%)	22 (55.0%)	
OR with sedation or general anesthesia	16 (84.2%)	3 (15.8%)		16 (66.7%)	8 (33.3%)	
The following statement best describes your experience with BalloonCatheter dilatation:			0.157			0.151
I am skeptical with regard to its added value	11 (64.7%)	6 (35.3%)		11 (55.0%)	9 (45.0%)	
I usually use it with primary probing if clinical picture suggests narrow duct rather than full obstruction	3 (100.0%)	0 (0.0%)		3 (75.0%)	1 (25.0%)	
I would use it only for failed and redo probing	1 (25.0%)	3 (75.0%)		1 (14.3%)	6 (85.7%)	
I would like to consider their use but not available where I practice	19 (73.1%)	7 (26.9%)		19 (57.6%)	14 (42.4%)	
The following statement best describes your experience with Dacryocystorhinostomy (DCR):			0.930			0.340
I have no experience doing it	24 (66.7%)	12 (33.3%)		24 (60.0%)	16 (40.0%)	
I do DCR. External is my preferred choice	8 (72.7%)	3 (27.8%)		8 (40.0%)	12 (60.0%)	
Yes, Endoscopic endonasal DCR is my preferred choice if available	2 (66.7%)	1 (33.3%)		2 (50.0%)	2 (50.0%)	
Yes, Non-endoscopic endonasal DCR	0 (0.0%)	0 (0.0%)		0 (0.0%)	0 (0.0%)	
Yes, Laser DCR	0 (0.0%)	0 (0.0%)		0 (0.0%)	0 (0.0%)	
A child presents to you with epiphora suggestive of CNLDO with no history of any intervention. When would you consider DCR for the primary procedure?			0.427			0.317
If age >3 years	4 (100.0%)	0 (0.0%)		4 (80.0%)	1 (20.0%)	
If age >4 years	3 (60.0%)	2 (40.0)		3 (50.0%)	3 (50.0%)	
If age >6 years (neglected cases)	3 (100.0%)	0 (0.0%)		3 (100.0%)	0 (0.0%)	
I would never do it as a primary intervention	12 (63.2%)	7 (36.8%)		12 (46.2%)	14 (53.8%)	
I would not do it myself but would consider for selected cases of failed probing	12 (63.2%)	7 (36.8%)		12 (50.0%)	12 (50.0%)	
The following statement best describes your experience regarding the child presenting with failed primary simple probing:			0.010			0.012
Repeat simple probing keeping in mind the age limit that I stick to with regard to stent placement	8 (57.1%)	6 (42.9%)		8 (44.4%)	10 (55.6%)	
Repeat probing with silicon tube stenting regardless of age	10 (50.0%)	10 (50.0%)		10 (37.0%)	17 (63.0%)	
Repeat probing with infracture of inferior turbinate ± silicon intubation	3 (100.0%)	0 (0.0%)		3 (75.0%)	1 (25.0%)	
Refer failed cases to oculoplastic and pediatric ophthalmologists	13 (100.0%)	0 (0.0%)		13 (86.7%)	2 (13.3%)	
The following statement best describes your experience with the child presenting with failed probing and stenting:			0.465			0.077
Redo probing with stenting	4 (57.1%)	3 (42.9%)		4 (40.0%)	6 (60.0%)	
Redo probing with stenting with infracture of inferior turbinate	4 (80.0%)	1 (20.0%)		4 (50.0%)	5 (50.0%)	
Redo with DCR done by myself	1 (33.3%)	2 (66.7%)		1 (14.3%)	6 (85.7%)	
Refer to a colleague who does DCR	25 (71.4%)	10 (28.6%)		25 (64.1%)	14 (35.9%)	

## Discussion

Despite the fact that most cases of CNLDO resolve spontaneously within the first year of life, some could persist beyond this date and cause huge distress to both child and family. Therefore, a proper therapeutic strategy for CNLDO is essential. Management lines of CNLDO include observation, medical, and surgical management.^20^,^21^ Massaging the lacrimal sac using Criggler’s technique is usually the first preferred management line. Kushner B^22^ reported that Criggler’s technique speeds resolution of CNLDO through increasing the hydrostatic pressure, which leads to rupture of the membranous obstruction in the valve of Hasner with high success rates. In our study, 62.7% of respondents (n = 52) recommended Criggler’s nasolacrimal duct massage for up to 12 months. Probing is usually considered the first intervention performed for CNLDO failed conservative measures.^10^ Despite the fact that the cure rates for early probing reach 78–100% during the first 12 months of age, these are no different to rates for spontaneous resolution up to 12 months of age.^11^,^23^ Repka et al^12^ reported that primary probing in children with CNLOD success rates were 78% overall and was 78% for subjects aged 6 to <12 months, 79% for subjects aged 12–36 months, and 56% for subjects aged 36–48 months. Also, they reported that success rates were lower in eyes operated in an office setting compared with in an operating theatre (ARR = 0.88 [95% CI = 0.80 to 0.96]). Furthermore, they found that success rates were lower in eyes of subjects with bilateral CNLDO (ARR = 0.88 [95% CI = 0.81 to 0.95]).^12^ Nevertheless, probing may injure the nasolacrimal duct epithelium leading to cicatricial fibrosis and thus preventing the spontaneous resolution of CNLDO.^11^ Resolution rates of second probing are largely reduced.^24^ Kakizaki et al^25^ reported that the cure rates of primary probing were 92% in the first year, 84.5% in the second year, 65% in the third year, and 63.5 in the fourth and fifth years of age. Furthermore, probing could be complicated with bleeding and canalicular obstructions, which occurs in 20% and 44% of cases, respectively.^25^ Hence, a “wait-and-see” approach with conservative measures is recommended to prevent such iatrogenic complications. Honavar et al^26^ recommended that early probing before 12 months of age should be withheld if symptoms such as dacryocystitis or severe blepharitis are absent.

There are several treatment options for CNLDO if a primary probing failed. These include inferior turbinate fracture, repeated probing, balloon catheter dilation, silicone tube intubation and dacryocystorhinostomy (DCR). Monocanalicular silicone intubation offers high success rates for CNLDO. Engel et al^15^ reported 97% success rate when performed in children younger than 24 months of age. However, bicanalicular silicone intubation has been used more frequently because it is generally more tolerated by the cornea.^27^ Andalib et al’s^28^ study showed no difference in outcomes between monocanalicular and bicanalicular stenting. In addition to that, they found that monocanalicular stenting is easier in tube removal without sedation in the office.^28^ In our study, monocanalicular stent was preferred by 42.2% of respondents (n = 27) while 31.3% (n = 20) preferred bicanalicular stent. Dacryocystorhinostomy is considered the last resort and usually indicated for failure of standard probing, balloon dilation and/or stents.^29^ External DCR is the more common modality; nonetheless, due to technological advances, endoscopic DCR has recently gained popularity.^30^ The success rates of DCR in children are high reaching 88–96% for external and 82–92% endoscopic approaches.^31^ However, continuous changing anatomy in children, poor-defined anatomy and extensive development of fibrosis have all been proposed as variables that limit surgical outcomes of DCR in children.^32^

Interestingly, untreated CNLDO has been identified as a risk factor for the development of amblyopia.^33^ Silbert et al^34^ reported 22% prevalence of amblyopia in patients with CNLDO. This could be explained that excessive tearing in CNLDO causes blurred vision and form-deprivation amblyopia during the sensitive period of visual development.^35^ In our study, only 37.3% of respondents (n = 31) regularly evaluated the refraction of a child presenting with epiphora suggestive of CNLDO. Thus, children with symptoms of CNLDO required a thorough eye examination including a cycloplegic refraction and subsequent follow-up to prevent amblyopia. This is especially critical for general ophthalmologists since they reported less practice to evaluate the refraction for a child with CNLDO.

This study is considered to be a guide to identify the preferred practice patterns of ophthalmologists regarding the management of CNLDO in order to be able to identify the knowledge gap and faulty practice that can be corrected with clear guidelines setting. In addition, to provide general ophthalmologists better insights regarding their practice for children with CNLDO. Limitations of the study are the small number of participants which may create a risk for selection bias as the data of who responded will be different from other who do not respond to the questionnaire. Moreover, these are considered self-reported practice preferences of ophthalmologists in Jordan and not the results of a randomized clinical trial. Thus, interpretation of our findings should be with caution and limited to what is more commonly accepted in the international levels without implying that less common practices are necessarily incorrect.

## Conclusions

Congenital nasolacrimal duct obstruction (CNLDO) is a common disorder among children. There is considerable variability in preferred practice patterns regarding the diagnosis and management of CNLDO in Jordan. Our findings highlight the gaps in optimum practices which need to be addressed for better management. Larger prospective studies are recommended to establish a consensus on the best approach for patients with CNLDO.

## Data Availability

All data generated or analyzed during this study are included in this article. Further enquiries can be directed to the corresponding author.
